# The prescription drug monitoring program in a multifactorial approach to the opioid crisis: PDMP data, Pennsylvania, 2016–2020

**DOI:** 10.1186/s12913-023-09272-3

**Published:** 2023-04-13

**Authors:** Jenna R. Adalbert, Amit Syal, Karan Varshney, Brandon George, Jeffrey Hom, Asif M. Ilyas

**Affiliations:** 1 Sidney Kimmel Medical College at Thomas Jefferson University, Philadelphia, PA USA; 2 Jefferson College of Population Health, Philadelphia, PA USA; 3 Deakin University School of Medicine, Geelong, VIC USA; 4 Philadelphia Department of Public Health, Philadelphia, PA USA; 5 Rothman Orthopaedic Institute Foundation for Opioid Research & Education, Philadelphia, PA USA

**Keywords:** Opioids, Opioid epidemic, Opioid policy, PDMP, Prescription opioids, Public health

## Abstract

**Background:**

Prescription opioids remain an important contributor to the United States opioid crisis and to the development of opioid use disorder for opioid-naïve individuals. Recent legislative actions, such as the implementation of state prescription drug monitoring programs (PDMPs), aim to reduce opioid morbidity and mortality through enhanced tracking and reporting of prescription data. The primary objective of our study was to describe the opioid prescribing trends in the state of Pennsylvania (PA) as recorded by the PA PDMP following legislative changes in reporting guidelines, and discuss the PDMP’s role in a multifactorial approach to opioid harm reduction.

**Methods:**

State-level opioid prescription data summaries recorded by the PA PDMP for each calendar quarter from August 2016 through March 2020 were collected from the PA Department of Health. Data for oxycodone, hydrocodone, and morphine were analyzed by quarter for total prescription numbers and refills. Prescription lengths, pill quantities, and average morphine milliequivalents (MMEs) were analyzed by quarter for all 14 opioid prescription variants recorded by the PA PDMP. Linear regression was conducted for each group of variables to identify significant differences in prescribing trends.

**Results:**

For total prescriptions dispensed, the number of oxycodone, hydrocodone, and morphine prescriptions decreased by 34.4, 44.6, and 22.3% respectively (*p* < 0.0001). Refills fluctuated less consistently with general peaks in Q3 of 2017 and Q3 of 2018 (*p* = 0.2878). The rate of prescribing for all opioid prescription lengths decreased, ranging in frequency from 22 to 30 days (47.5% of prescriptions) to 31+ days of opioids (0.8% of prescriptions) (*p* < 0.0001). Similarly, decreased prescribing was observed for all prescription amounts, ranging in frequency from 22 to 60 pills (36.6% of prescriptions) to 60–90 pills (14.2% of prescriptions) (*p* < 0.0001). Overall, the average MME per opioid prescription decreased by 18.9%.

**Conclusions:**

Per the PA PDMP database, opioid prescribing has decreased significantly in PA from 2016 to 2020. The PDMP database is an important tool for tracking opioid prescribing trends in PA, and PDMPs structured similarly in other states may enhance our ability to understand and influence the trajectory of the U.S. opioid crisis. Further research is needed to determine optimal PDMP policies and practices nationwide.

## Introduction

Today’s opioid epidemic in the United States (U.S.) is a public health crisis, instigated partly by healthcare provider overprescribing [1]. While the U.S. constitutes merely 5% of the global population, the nation consumes 80% of the world’s entire opioid supply and 99% of hydrocodone [2]. Provider fear of undermanaging patient pain and a lack of opioid prescribing guidelines drive excessive prescribing, a practice that confers significant risks [3, 4]. Extensive opioid prescription lengths increase the risk of long-term opioid dependence for patients, with the additional consequence of an increased number of opioid pills available for diversion to unintended recipients in the community [5]. Over the past two decades, increasing rates of opioid prescriptions and opioid overdoses have quadrupled in parallel [6] with opioid overdose prominently ranking as a leading cause of injury-related death in the U.S. [7]. While opioid-related overdose deaths are now due predominantly to illicitly-manufactured fentanyl, and to a lesser extent heroin, prescription opioids remain an important contributor to today’s crisis and the development of opioid use disorder for opioid-naïve individuals [8, 9].

In response to the severity of the U.S. opioid crisis, interventions targeted at the prescriber-level have included initiatives such as opioid management recommendations from government agencies [10] and the publication of specialty- and procedure-specific prescribing guidelines [11]. In terms of measuring the efficacy of these opioid stewardship interventions and monitoring prescribing practices, a prominent legislative action has been the development of state-specific prescription drug monitoring programs (PDMPs). While PDMP requirements for prescriber consultation and dispensary reporting differ between states, the general goal of PDMP implementation is to track controlled substance prescribing through a statewide database [12]. For opioids specifically, the potential of PDMPs to reduce the harms of prescribed opioids exists in their ability to identify risky healthcare provider prescribing behaviors and alert providers to patients with multiple opioid prescriptions or co-prescribed controlled medications that may increase overdose risk [13]. While studies assessing PDMP success at harm reduction per these metrics have reported variable outcomes [14, 15], PDMP data may be a valuable tool for accurately visualizing longitudinal trends in opioid prescribing.

Although the opioid epidemic is a nationwide population health issue, the state of Pennsylvania (PA) consists of a particularly vulnerable demographic. In 2016, the state was third in the nation for opioid overdose morbidity and mortality [16, 17]. There were 4314 deaths in PA that involved an opioid in 2020, comprising 85% of all PA overdose deaths that year [17]. This number was a 16% increase in opioid-related deaths compared to 2019 (3728) and a 7 % decrease from 2017 (4645). Prescription opioids contribute greatly to opioid-related morbidity as well: nationally, after only a single day’s consumption of an opioid prescription, the rate of persistent opioid use is 6% at 1 year, and escalates to 13.5% if initial prescription duration reaches a minimum of 7 days [18]. With dependence or misuse initiated by prescription opioids, individuals may be susceptible to transition to a more potent illicit opioid and a subsequently increased overdose risk [19, 20].

Given the potential of PDMPs to serve as an important tool for tracking opioid distribution and quantifying areas of significant opioid distribution per population in states with high opioid morbidity and mortality, the purpose of our study was two-fold. First, we aimed to publicly present the trends of the three most commonly prescribed opioids in PA – oxycodone, hydrocodone, and morphine – from August 2016 to March 2020, as reported by the PA PDMP. In addition to presenting these prescribing trends, we also aimed to provide data on prescription lengths, quantities, and average morphine milliequivalents (MME) for all 14 opioid medications recorded by the PA PDMP so that this data may be utilized in further studies and compared to prescribing trends in other states for distribution mapping purposes. The second aim of our study was to discuss PA’s approach to regulating prescription opioids through the PDMP from August 2016 to March 2020. During the time period that coincides with our data, several key legislations were enacted to promote opioid stewardship that may offer harm reduction insight when compared to interventions in other states [21–23]. In the context of our findings, we then reviewed and explored the utility of PDMPs in a multifactorial approach to opioid harm reduction.

## Methods

This retrospective, cross-sectional study using de-identified, aggregate prescription data was determined exempt by the Thomas Jefferson University Institutional Review Board.

### Data source

Monitored by the PA Department of Health (DOH), the PA PDMP is an electronic, statewide database that obtains information on all controlled substance (Schedule II-V) prescriptions filled at a dispensary [21]. For opioids specifically, the PA DOH collects and records several opioid prescribing metrics reported through the statewide PDMP as state- and county-level data. Prior to October 2014, the Office of the Attorney General operated the PA PDMP with required reporting limited to Schedule II substances [21]. PA legislature transferred operations and development of the statewide PDMP to the PA DOH following Act 191 of 2014 [22] which led to the official launch of a new PDMP by the PA DOH in August 2016. Starting January 1, 2017, all licensed dispensers in PA were mandated by the state government to report all dispensed Schedule II-V prescriptions to the PA PDMP [23]. This legislation was accompanied by the requirement that all licensed prescribers with authorization to distribute, dispense, or administer controlled substances in PA register for the PDMP and query its information at select times to inform prescribing decisions [23].

### Data metrics

For the purposes of this study, the PA DOH provided de-identified state-level opioid prescription data summaries recorded by the PDMP for each calendar quarter from August 2016 (the official launch date of data collection) through March 2020. The dataset used in this study came from a database that provides statewide and countywide surveillance of opioid prescribing. As the dataset used statewide and countywide data, calculation of sample sizes was not required.

Data summaries included prescribing metrics for 14 different prescription opioids: belladonna-opium, codeine, fentanyl, hydrocodone, hydromorphone, levorphanol, meperidine, methadone, morphine, oxycodone, oxymorphone, sufentanil, and tapentadol. Buprenorphine was excluded from all provided metrics. Metrics reported per quarter included the total number of each type of opioid prescribed, the total number of each type of opioid prescription refill, the length of these prescriptions categorized into five groups (3 days or less, 4–7 days, 8–21 days, 22–30 days, 31 days or more), and the quantity of these prescriptions categorized into four groups (21 pills or less, 22–60 pills, 60–90 pills, 90 pills or more). The average prescription morphine milliequivalent (MME) calculated from all opioids prescribed per quarter was also provided.

### Data analysis

From the metrics of total number of prescription opioids and their respective refill data, we selected to analyze oxycodone, hydrocodone, and morphine. During any quarter from August 2016 to March 2020, these three prescription opioids comprised approximately 90% of opioids prescribed by healthcare providers in PA and represent a comprehensive demographic of prescribers by type and geography [24]. The remaining analyzed metrics included all 14 opioid prescription variants. Data were extracted, tabled, and graphed for longitudinal review of prescribing trends. Simple linear regression was conducted to form best-fit lines and to compare if there were significant differences between slopes, where quarter & year was the predictor variable and total number of prescriptions was the outcome variable. An F-test was run to determine if there was a difference in slopes for lines of best-fit for each opioid. *P*-values, as well as F-test values were reported. Graphs were created and simple linear regression of data was conducted using GraphPad Prism v.9.0.0 for Windows (GraphPad Software, San Diego, CA).

## Results

Table 1 is a comprehensive report of the five prescription opioid metrics, as provided by the PA DOH for each calendar quarter (August 2016 to March 2020), recorded by the PA PDMP.

**Table 1:** Five prescription opioid metrics reported for each calendar quarter (August 2016 to March 2020) by the PA PDMP

	Q3: 2016	Q4: 2016	Q1: 2017	Q2: 2017	Q3: 2017	Q4: 2017	Q1: 2018	Q2: 2018	Q3: 2018	Q4: 2018	Q1: 2019	Q2: 2019	Q3: 2019	Q4: 2019	Q1: 2020	Total
**Total Prescriptions**																
Oxycodone	961,172	926,393	889,615	876,227	839,694	829,178	780,692	768,219	737,711	730,145	689,365	683,899	672,278	665,178	630,888	**11,680,654**
Hydrocodone	662,695	635,409	603,834	578,103	544,679	528,251	499,922	483,970	454,569	448,367	421,675	414,409	400,645	389,044	366,966	**7,432,538**
Morphine	135,983	135,267	135,431	135,724	131,504	131,524	127,520	124,100	120,833	118,724	112,908	112,601	110,061	108,636	105,668	**1,846,484**
**Number of Refills**																
Oxycodone	262	542	814	751	1079	1003	851	853	1099	854	626	667	817	780	685	**11,683**
Hydrocodone	161	306	355	319	392	323	338	355	591	481	473	409	387	293	306	**5489**
Morphine	557	524	682	687	683	621	505	301	407	361	300	371	355	432	481	**7267**
**Length of Prescriptions**																
≤3 days	319,335	286,719	281,422	275,355	264,769	267,280	251,501	259,201	248,127	245,350	239,943	244,412	241,086	230,447	211,834	**3,866,781**
4–7 days	320,389	311,816	297,548	281,760	266,245	264,060	257,355	246,218	235,285	237,871	235,133	226,706	218,625	223,112	210,358	**3,832,481**
8–21 days	386,075	376,637	353,965	341,590	317,966	316,222	291,670	275,322	254,290	246,428	221,164	216,074	208,830	204,808	193,295	**4,204,336**
22–30 days	898,432	883,395	851,868	845,278	810,498	777,430	733,829	718,186	692,614	681,180	631,947	626,326	612,760	598,172	575,078	**10,936,993**
31+ days	17,173	16,586	15,320	15,116	14,259	13,833	11,446	11,205	10,610	10,050	7952	7593	7726	7766	7394	**174,029**
**Prescription Quantities**																
< 21 pills	483,247	440,366	429,994	426,612	414,563	412,803	391,440	398,893	386,402	382,802	374,481	378,264	373,988	360,837	338,537	**5,993,229**
22–60 pills	717,408	706,164	670,870	651,618	613,652	606,814	575,273	553,986	524,599	516,549	481,819	472,732	457,476	455,179	428,840	**8,432,979**
60–90 pills	267,419	263,492	253,923	250,236	241,086	234,246	220,367	215,999	206,326	203,154	187,990	185,963	182,245	178,894	172,176	**3,263,516**
90+ pills	473,329	465,130	445,335	430,632	404,436	384,962	358,721	341,254	323,599	318,372	291,844	284,151	275,315	269,394	258,405	**5,324,879**
**Morphine Milliequivalents**																
Average MME	68.06	65.86	63.49	62.65	62.73	60.98	60.33	59.07	58.38	57.84	56.67	55.31	55.02	55.11	55.23	**59.78**

### Total prescriptions

**Fig. 1:**
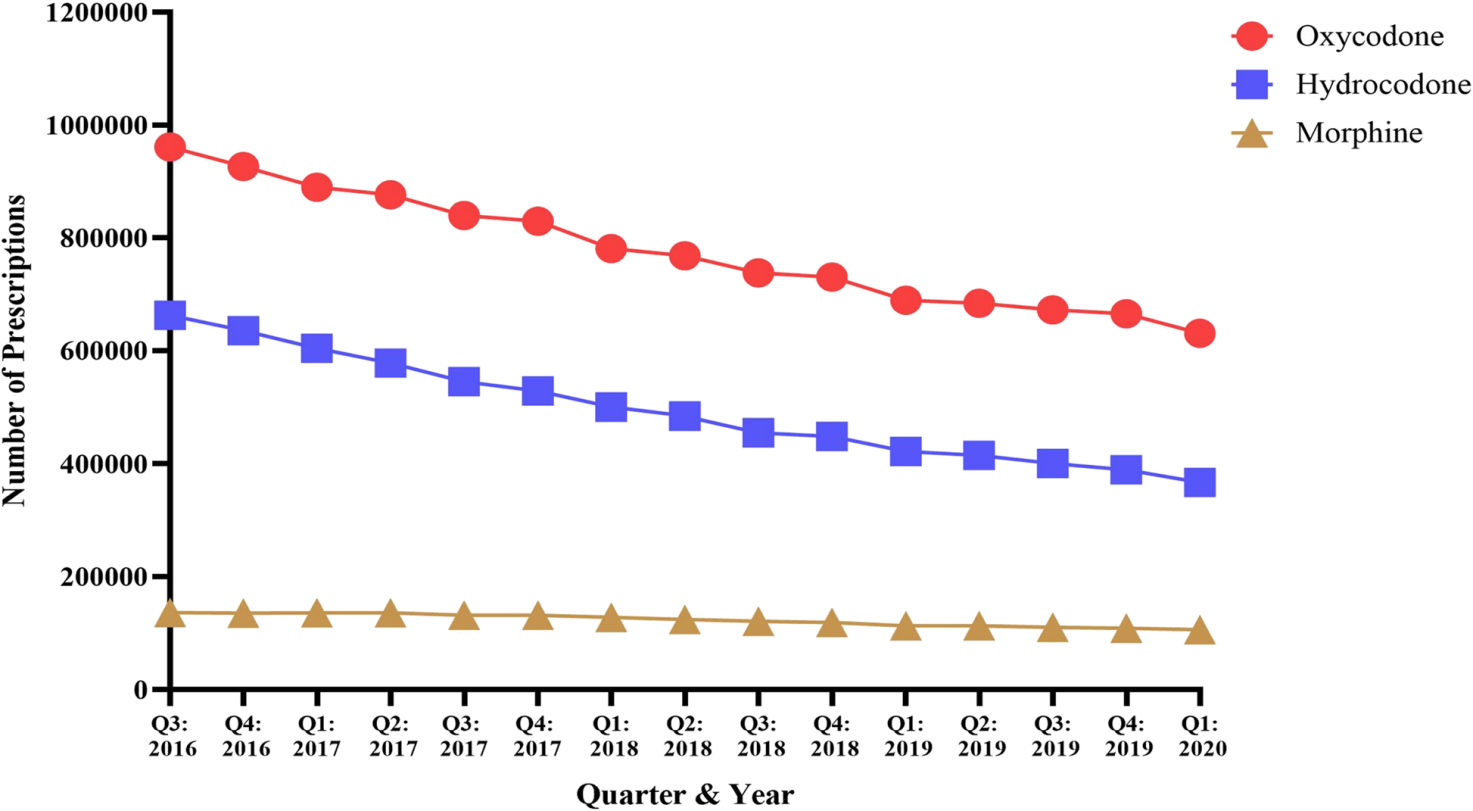
Total number of oxycodone, hydrocodone, and morphine prescriptions dispensed in PA from August 2016 to March 2020 per the PDMP

Overall, a total of 20,959,676 oxycodone, hydrocodone, and morphine prescriptions were dispensed in PA from the third quarter (Q3) of 2016 to the first quarter (Q1) of 2020. Of these prescriptions, 11,680,654 were oxycodone, 7,432,538 were hydrocodone, and 1,846,484 were morphine (Table 1). For oxycodone, prescriptions ranged from 961,172 in Q3 of 2016 to 630,888 in Q1 of 2020, displaying a consistent decline in prescribing (34.4% decrease) (Fig. 1). Hydrocodone and morphine demonstrated similar downward prescribing trends with hydrocodone prescriptions ranging from 662,695 in Q3 of 2016 to 366,966 in Q1 of 2020 (44.6% decrease), and morphine prescriptions ranging from 135,983 in Q3 of 2016 to 105,668 in Q1 of 2020 (22.3% decrease) (Fig. 1). Regression analysis indicated that the slopes of oxycodone, hydrocodone, and morphine total prescription amounts were different (*p* < 0.0001; F = 249.5, DFn = 2, DFd = 39).

### Number of refills

**Fig. 2:**
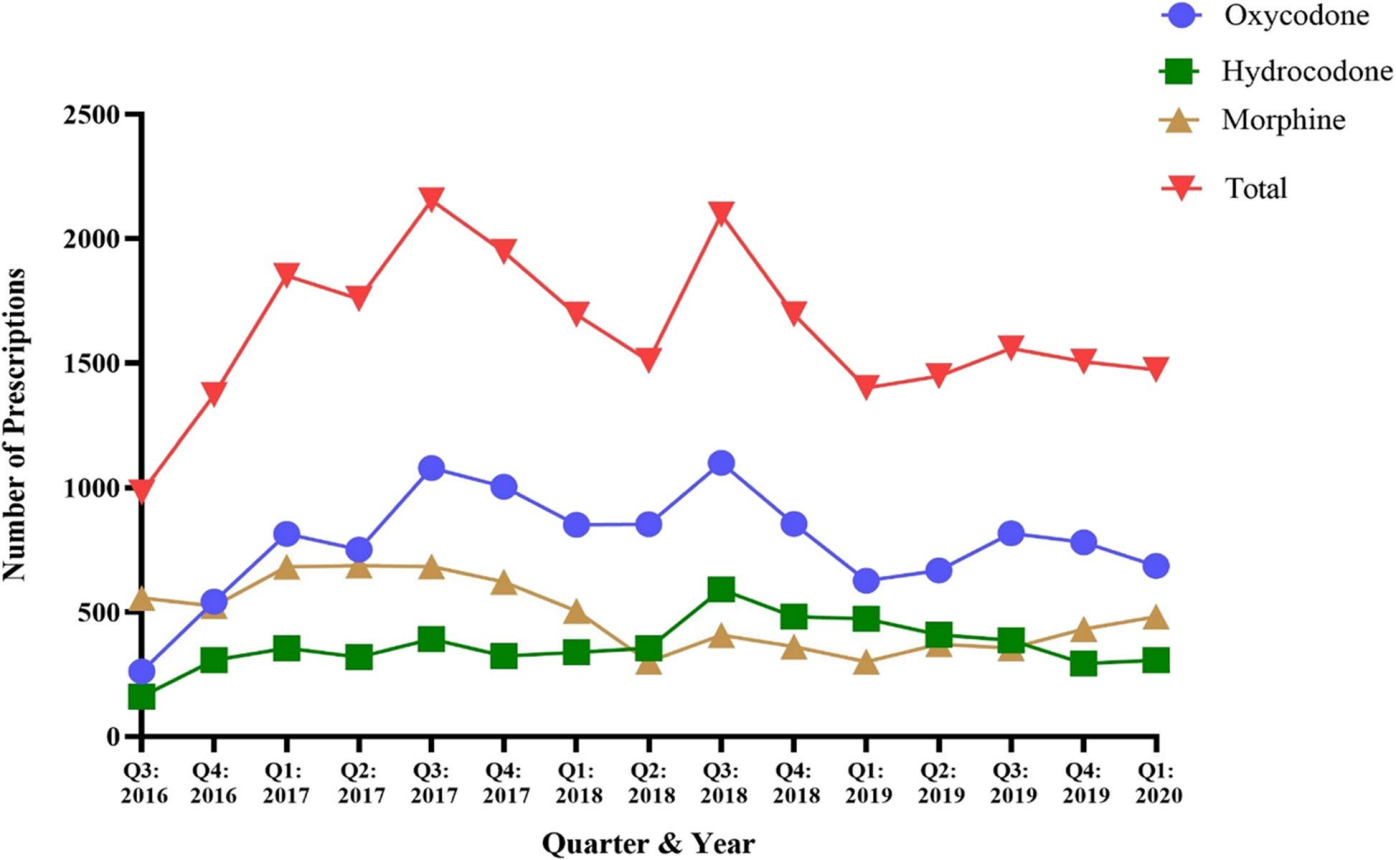
Total number of oxycodone, hydrocodone, and morphine refills dispensed in PA from August 2016 to March 2020 per the PDMP

Following an initial opioid prescription, a total of 11,683 oxycodone, 5489 hydrocodone, and 7267 morphine prescription refills were dispensed in PA from Q3 of 2016 to Q1 of 2020 (Table 1). There was a wide range of oxycodone prescription refills, from a maximum of 1099 refills in Q3 of 2018 to a minimum of 262 in Q3 of 2016 (Fig. 2). For hydrocodone, prescription refills reflected a more consistent pattern with a minimum of 161 in Q3 of 2016 that reached a maximum of 591 in Q3 of 2018, with subsequent quarters reflecting a relatively downward trend (Fig. 2). Morphine prescription refills reached a peak of 687 in Q2 of 2017, with a low of 300 in Q1 of 2019 and depicted a relatively variable prescription refill pattern (Fig. 2). To create a general opioid prescription refill trend, consolidation of oxycodone, hydrocodone, and morphine prescriptions refills revealed a trendline with notable peaks in Q3 of 2017 and Q3 of 2018 (Fig. 2). Regression analysis demonstrated that the slopes of oxycodone, hydrocodone, and morphine refills were not different (*p* = 0.2878; F = 1.290, DFn = 3, DFd = 52).

### Length of prescriptions

**Fig. 3:**
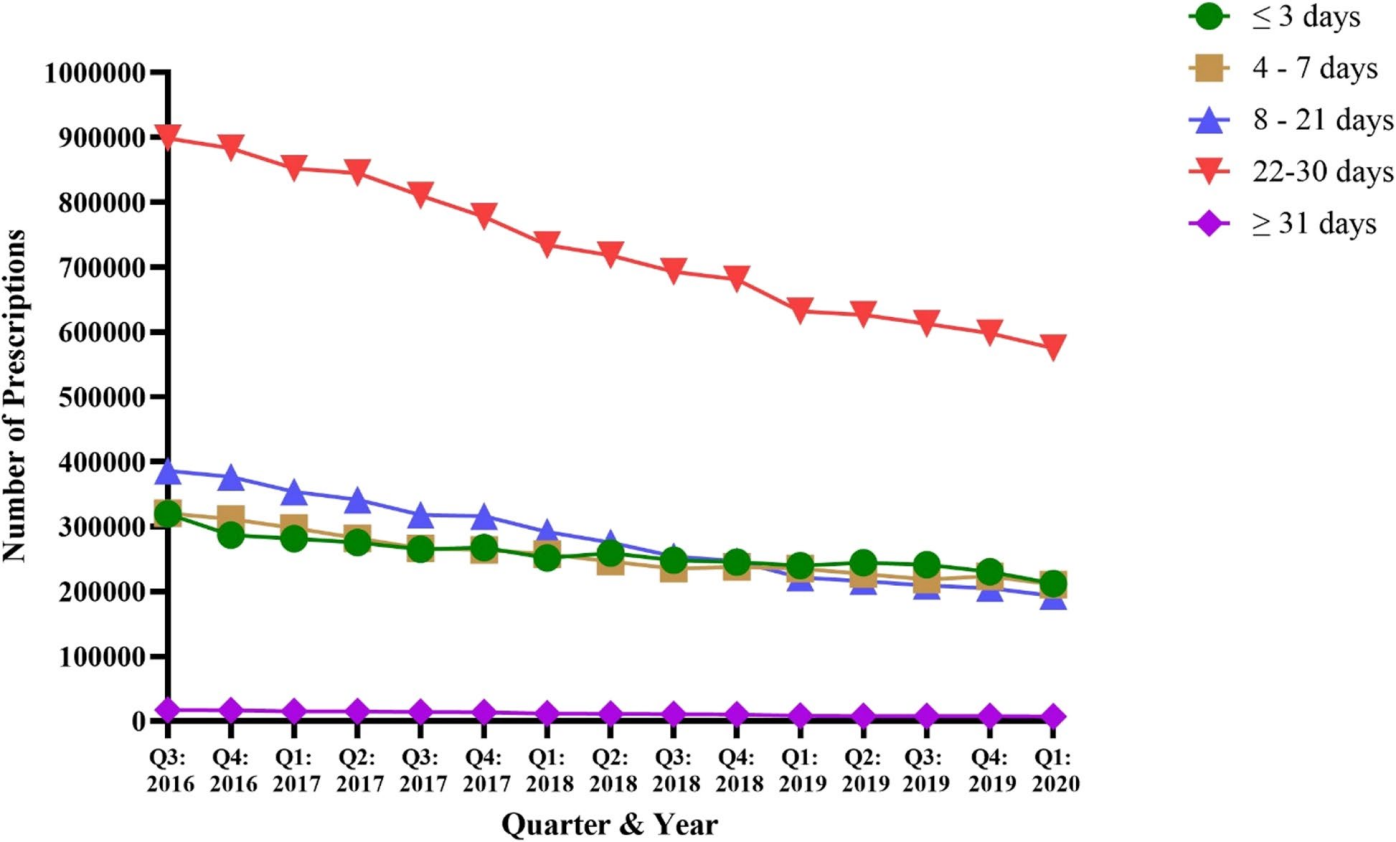
Number of opioid prescriptions by length of prescription days dispensed in PA from August 2016 to March 2020 per the PDMP

For all opioids prescriptions dispensed in PA from Q3 of 2016 to Q1 of 2020, 3,866,781 (16.8%) were prescribed for 3 days or less, 3,832,481 (16.6%) were 4–7 days, 4,204,336 (18.3%) were 8–21 days, 10,936,993 (47.5%) were 22–30 days, and 174,029 (0.8%) were 31 or more days in length (Table 1). In general, all opioid prescription lengths decreased in prescribing frequency from Q3 of 2016 to Q1 of 2020 (Fig. 3). Opioid prescriptions dispensed for 22–30 days were by far the most common prescription length, ranging from 898,432 prescriptions in Q3 of 2016 to 575,078 in Q1 of 2020 (36.0% decrease) (Fig. 3). The least common opioid prescription length was 31 or more days, with a range of 17,173 prescriptions dispensed in Q3 of 2016 and 7394 in Q1 of 2020 (56.9% decrease) (Fig. 3). Regression analysis showed that the slopes of the five opioid prescription lengths were different (*p* < 0.0001; F = 282.8, DFn = 4, DFd = 65).

### Prescription quantities

**Fig. 4:**
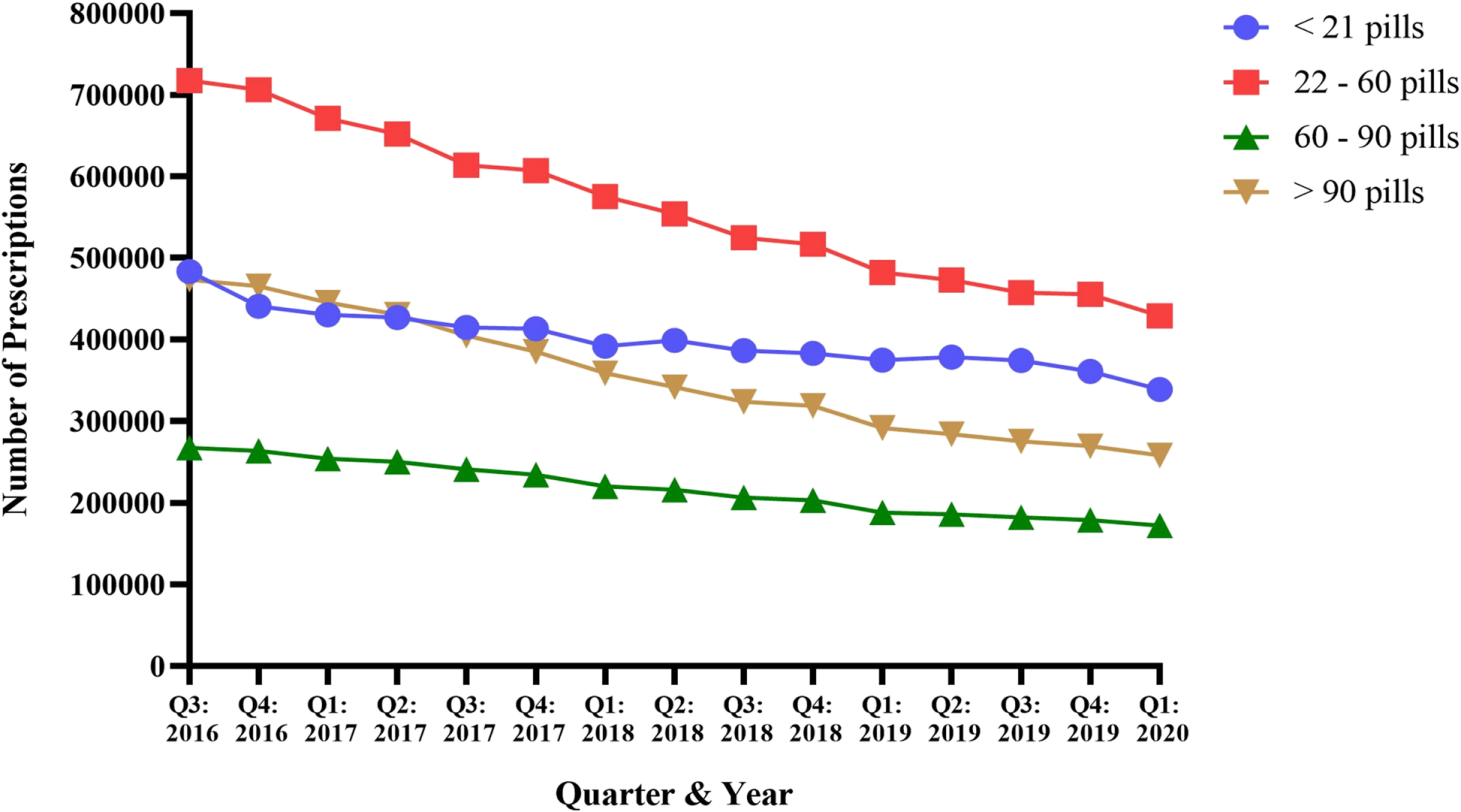
Number of opioid prescriptions by quantity of opioid pills dispensed in PA from August 2016 to March 2020 per the PDMP

In terms of individual opioid prescription amounts dispensed in PA from Q3 of 2016 to Q1 of 2020, 5,993,229 (26.1%) prescriptions consisted of 21 pills or less, 8,432,979 (36.6%) consisted of 22–60 pills, 3,263,516 (14.2%) consisted of 60–90 pills, and 5,324,879 (23.1%) consisted of 90 pills or more (Table 1). Overall, a decrease in prescribing was identified for all prescription opioid quantities from Q3 of 2016 to Q1 of 2020 (Fig. 4). Prescriptions consisting of 22–60 pills were by far the most commonly dispensed, trending downwards from 717,408 prescriptions in Q3 of 2016 to 428,840 in Q1 of 2020 (40.2% decrease) (Fig. 4). Conversely, prescriptions consisting of 60–90 pills were the least commonly prescribed, decreasing from 267,419 prescriptions dispensed in Q3 of 2016 to 172,176 in Q1 of 2020 (35.6% decrease) (Fig. 4). Regression analysis indicated that the slopes of the four opioid prescription amounts were different (*p* < 0.0001; F = 126.7, DFn = 3, DFd = 52).

### Average MMEs

**Fig. 5:**
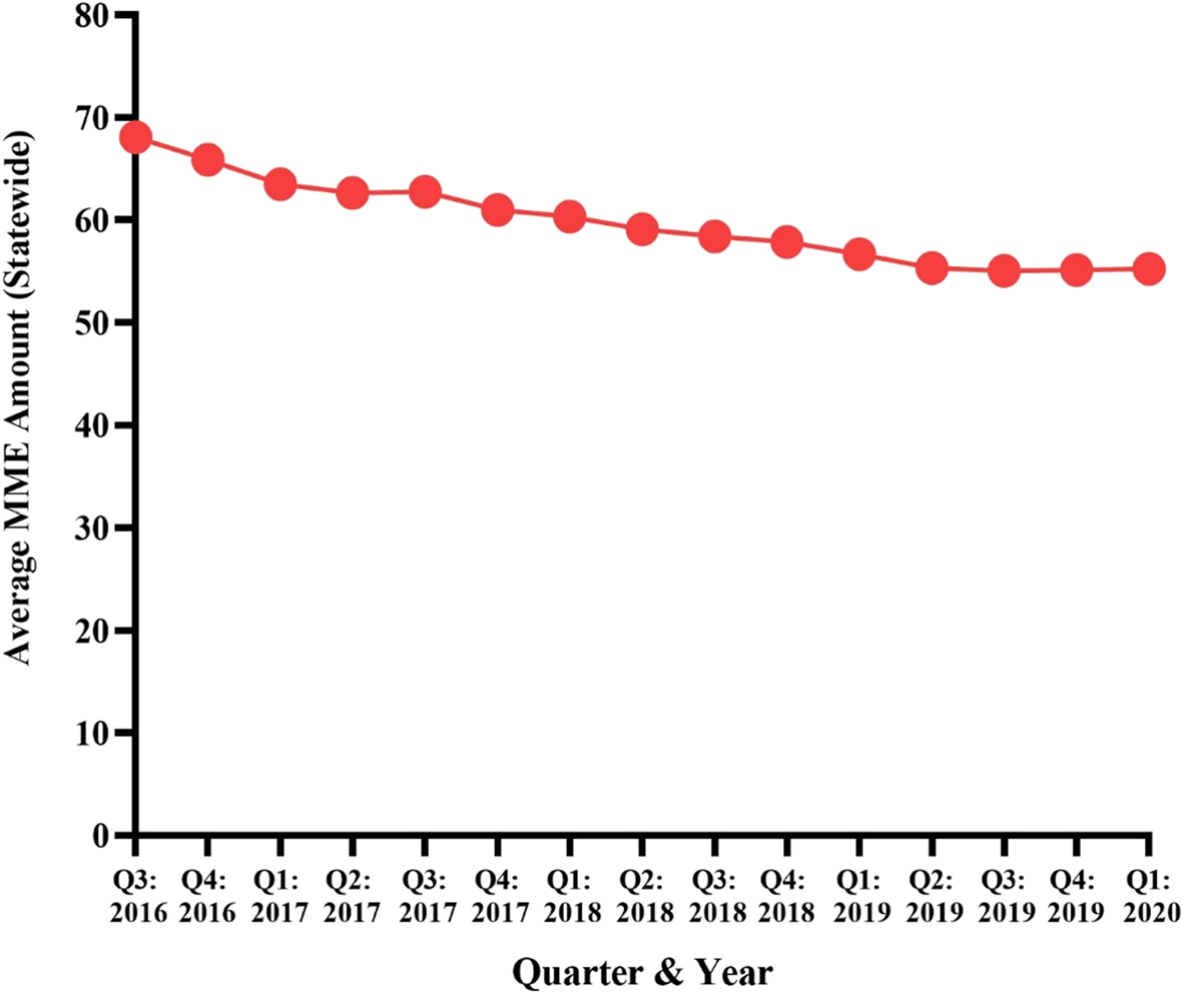
Average MME amount per opioid prescription dispensed in PA from August 2016 to March 2020 per the PDMP

Overall, the average MME amount per opioid prescription dispensed in PA from Q3 of 2016 to Q1 of 2020 was 59.78 (Table 1). Average MMEs per opioid prescription trended downwards from 68.06 in Q3 of 2016 to 55.23 in Q1 of 2020 (18.9% decrease) (Fig. 5).

## Discussion

Our longitudinal review of the PA PDMP opioid prescription data demonstrates that total prescriptions, length of prescriptions, prescription pill quantities, and average MMEs have decreased significantly in the state of PA. Per our complete annual data available following attainment from the PA DOH from 2017 to 2019, the rate of prescribing in PA was 53.7 per 100 population in 2017, 46.2 per 100 population in 2018, and 40.7 per 100 population in 2019 [25]. To understand PA’s prescribing trends in the context of available nationwide data adjusted per population and provided by the CDC, the rate of opioids dispensed nationwide has declined progressively since 2012, with 59.0 per 100 population in 2017, 51.4 per 100 population in 2018, and 46.7 per 100 population in 2019 [26]. During this time, PA’s overall state population has remained relatively constant with a total population of 12,788,468 individuals in 2016, 12,794,679 in 2017, 12,809,107 in 2018, 12,798,883 in 2019, and 12,989,625 in 2020 [25]. To provide context on the ranges of prescribing that exist between states, Alabama dispensed 108.8 and District of Columbia 28.4 per 100 population in 2017, Alabama dispensed 97.5 and District of Columbia 25.0 per 100 population in 2018, and Alabama dispensed 85.8 and Hawaii dispensed 30.3 per 100 population in 2019 [26]. Comparatively, PA’s 2017–2019 opioid prescribing rates fall within the middle third of this national prescribing range, reflecting the overall decrease in prescribing trends observed at the national level. It is also crucial to mention that the PA PDMP does not record data on tramadol, a combination drug composed of opioid and serotonin/norepinephrine reuptake inhibitor that was not recognized as a Schedule IV controlled substance until 2014 [27]. While tramadol is considered an effective analgesic with a low potential for abuse, it has been associated with a higher risk of death in comparison to other common analgesics such as naproxen or diclofenac [28]. We emphasize this point given the discrepancy between CDC PA prescribing rates and our PA PDMP data – when adjusted for tramadol, PA opioid prescribing trends continued to reflect a consistent decrease, but rates were increased to 58.3 per 100 population in 2017, 49.9 per 100 population in 2018, and 47 per 100 population in 2019 [26].

While this decrease in national opioid prescribing rates is attributed to awareness engendered by the opioid epidemic and interventions at federal and state levels targeting different levels of prevention [29, 30], the instillment of state PDMPs and mandates accompanying their use have been a key component of the U.S. approach to opioid harm reduction. The transition of PA PDMP operations to the PA DOH [21] in August of 2016 was accompanied by several important measures passed by PA legislature targeted at reducing harm from prescription opioids specifically. Designed to enhance the rigor of the PA PDMP, this legislation focused on restricting opioid prescription amounts and refills: Act 126 of 2016 [31] established a list of opioid initiation requirements and set a seven-day limit on opioid prescriptions for minors, while Act 122 of 2016 [32] set a seven-day limit and banned refills on opioid prescriptions written by hospital emergency department (ED) and urgent care center providers. To complement these restrictions, PDMP-specific legislative changes focused on enhanced reporting of controlled substance prescribing and increased provider consultation of the database. Specifically, Act 124 of 2016 [33] mandates that providers consult patient prescription records in the PDMP prior to every opioid or benzodiazepine prescription and refill, amending the previous requirement of a single consult upon initial prescription or at the prescriber’s judgement. This Act also amended dispenser requirements, with pharmacies required to submit dispensed opioid prescription data to the PDMP within 24 hours, in contrast to the previous 72-hour requirement. Overall, these legislative changes focused on several fundamental areas for prescription opioid harm reduction in the PA healthcare system. At the provider level, setting prescribing limits for vulnerable populations such as children or patients with brief provider contact in urgent and emergent settings is valuable for both protecting these groups and physically limiting the number of opioids dispensed into the community. Similarly, requiring regular provider consultation of the PDMP informs responsible prescribing and may reduce the overdose risk that accompanies multiple opioid prescriptions or opioid and benzodiazepine co-prescribing [34]. At the dispensary level, promptly inputting opioid data supports these provider level harm reduction measures and eliminates “doctor shopping” in which patients may surreptitiously visit numerous providers for multiple opioid prescriptions [35].

Regarding the nationwide effects of the emergence of PDMPs on opioid prescriptions, Bao et al. analyzed national ambulatory care data during the emergence of PDMPs from 2001 to 2010 and found that PDMP implementation was associated with a 30% reduction in the rate of Schedule II opioid prescribing [36]. Importantly, this reduction of opioids dispensed into the community was immediate and sustained through the second and third years following PDMP launch [36]. In the context of other states that have similarly increased PDMP rigor, our findings are generally consistent with the decrease in opioid prescribing that follows this type of legislative action [37–41]. Per our PA PDMP data and net values calculated from Table 1, the largest decreases in prescribing between 2016 and 2020 were the number of 90+ pills being prescribed (45.41% decline), 8–21 day length prescriptions (49.33% decline), and 31+ day length prescription (56.94% decline). Additionally, it is important to denote that during this four-year period, the number of refills of oxycodone increased (161.45% increase), as did the number of refills for hydrocodone (90.06% increase); while the number of morphine refills did instead decrease, it decreased less compared to any other metric analyzed in this study (13.65% decline). While primary opioid prescription trends have decreased consistently per the PA PDMP, the total number of prescription refills of oxycodone and hydrocodone demonstrates an increasing trend, which contrasts the findings of decreased refills following increased PDMP rigor in other states [37, 40]. Importantly, this constitutes a necessity for further research to understand the rationale for this increase in PA. Monitoring for all metrics, but especially refill totals, will be integral in future opioid surveillance in order to effectively guide policy enactment (Table 2).

**Table 2:** Summary of opioid prescribing in PA between 2016 and 2020 as per the PDMP and associated data

**Summary of Findings**			
*The PA PDMP has highlighted that across nearly every measure, prescriptions of oxycodone, hydrocodone, and morphine have declined between 2016 and 2020 in PA.*			
*High prescription quantities (90+ pills) and long length of prescriptions (31+ days) had the largest decline over this period, with a 45.41% decrease and 56.94% decrease respectively.*			
*These findings highlight that legislative measures, alongside multimodal pain treatments and preventive interventions, have been effective at lowering rates of opioid prescribing with monitoring of prescriptions by the PDMP integral in the tracking progress.*			
*Death attributed to opioid overdose has similarly shown a decrease over this period, but further investigation is required to understand what measures have effectively resulted in this change.*			
*PDMP program utility is evident for tracking prescriptions, but further research is needed to delineate its overall effectiveness in helping to lower total opioid-related deaths and crimes.*			
**Prescription metric**	**Starting total (at Q3: 2016)**	**Final total (at Q1: 2020)**	**% Net change from 2016 to 2020**
*Total number of prescriptions*			
Oxycodone	961,172	630,888	−34.36
Hydrocodone	662,695	366,966	−44.63
Morphine	135,983	105,668	−22.29
*Number of refills*			
Oxycodone	262	685	+ 161.45
Hydrocodone	161	306	+ 90.06
Morphine	557	481	−13.65
*Length of prescriptions*			
≤3 days	319,335	211,834	−33.66
4–7 days	320,389	210,358	−34.34
8–21 days	386,075	193,295	−49.33
22–30 days	898,432	575,078	−35.99
31+ days	17,173	7394	−56.94
*Prescription quantities*			
< 21 pills	483,247	338,537	−29.95
22–60 pills	717,408	428,840	−40.22
60–90 pills	267,419	172,176	−35.62
90+ pills	473,329	258,405	−45.41
*Average morphine milliequivalents*	68.06	55.23	−18.51

While the PA PDMP mandates are an important part of PA’s approach to opioid harm reduction, we emphasize that these changes in prescription opioid regulations in PA have been accompanied by initiatives targeted at patients particularly vulnerable to opioid overdose risk as part of a heterogenous approach. In November 2016, the Drug Enforcement Agency 360 Initiative was launched to combat the severity of opioid misuse and overdose in Pittsburgh, PA [24]. Using Pittsburgh as a pilot city prior to national implementation, the 360 Strategy brought together experts in substance misuse disorder and prevention to provide resources for educating youth about the consequences of opioid misuse, with the long-term goal of reducing overdose in the future [24]. This initiative focused on establishing relationships between community partners, treatment providers, educators, and policymakers, and currently fosters opioid misuse-related information sharing, integrated strategies, and resource discussion between these entities [24]. Additionally, the PA Centers of Excellence (COE) established a network of 45 facilities across PA that ensures individuals with opioid use disorder (OUD) have access to integrated and coordinated care and works to facilitate care for individuals with OUD who receive coverage through Medicaid [24]. In tandem with prescribing regulations, these types of initiatives create a supportive network to prevent the development of OUD and protect patients with the goal of a healthy and sustained recovery (Table 3).

**Table 3:** Opioid-related legislative measures/initiatives adopted in PA to address the opioid crisis

Name of Legislation/Initiative	Year adopted/ initiated	Summary of Purpose
Act 191 (PA Legislature)	2014	Requires monitoring Schedule II through Schedule V controlled substances; the PA DOH has become responsible for the development and maintenance of the new PDMP system [22].
Act 126 (PA Legislature)	2016	Restricts total opioid prescription amounts and refills [31].
Act 122 (PA Legislature)	2016	Establishes a list of opioid initiation requirements and creates a limit of 7 days for prescribing opioids to minors [32].
Act 124 (PA Legislature)	2016	Mandates that providers consult patient prescription records in the PDMP prior to prescribing and refilling every opioid/benzodiazepine; amends dispenser requirements to submit data to PDMP within 24 hours [33].
Drug Enforcement Agency 360 Initiative	2016	Provision of resources to educate youth about the dangers of opioid usage; developing relationships and discussions between various entities in order to lower rates of opioid overdoses; creating networks to prevent opioid use disorder (OUD) and protect those who have OUD [24].
PA Centers of Excellence Network	2016	Ensures that individuals with OUD receive coordinated and integrated care, with coverage through Medicaid; creates networks to prevent opioid use disorder (OUD) and protect those who have OUD [24].

Although available data on overdose deaths in PA attributed to prescription opioids show a simultaneous decrease congruent with our PDMP data trends starting in Q4 of 2016 [42], we caution any assumption that legislative action and enhanced PDMP rigor alone are sufficient to reduce the morbidity and mortality from prescription opioids. The PA PDMP’s association with decreased overdose deaths during our study period has likely been amplified by integration with interventions such as enhanced provider education focused on reduced opioid prescribing, increased use of multimodal pain treatments, and other key aforementioned initiatives. While the enactment of PDMP mandates may be associated with decreased prescribing, the literature supporting PDMP utility in opioid harm reduction at the population level is mixed. Multiple studies have shown no significant reductions in opioid-related deaths or crime rates following the implementation of a PDMP [15, 43]. Recently, a systematic review by Puac-Polanco et al. found that PDMPs with mandatory consultation policies were associated with reductions in prescribing behaviors, diversion outcomes, hospital admissions, substance-use disorders, and mortality rates [44]. Importantly, they identified that inconsistencies in the current PDMP evidence base were due to the diversity of analytical approaches across studies and heterogeneity in state PDMP policies [44]. This heterogeneity of state PDMPs creates barriers and limitations to effective PDMP use by the U.S. healthcare system. While a national PDMP database has been recognized as crucial for addressing opioid misuse activities that cross state lines, discrepancies in PDMP legislation related to consultation is a limitation to interstate data sharing [45]. PDMP design is another prominent barrier to their effectiveness in practice with providers in multiple states reporting difficulties with the lack of intuitive formatting and the time-consuming nature of data [46–49]. This suggests an important role for technical improvement in PDMP platform access for providers – enhanced ease of use and efficiency may advance the utility of PDMPs in the clinical environment and further clarify their true effectiveness in harm reduction at the population level when accompanied by legal mandates.

### Limitations

To our knowledge, this study is the first to directly report and analyze statewide prescribing trends using the PA PDMP data in the context of legislative PA PDMP initiatives via a multi-database review with PubMed, Scopus, Ovid MEDLINE, CINAHL and ScienceDirect. However, we warrant caution in drawing definitive conclusions from this data – rather, the effectiveness of the PDMP and related legislation should be considered in the context of multiple interventions. Other initiatives such as increased provider opioid education and awareness occurring amidst today’s opioid crisis have likely impacted prescribing trends, hence our emphasis that this significant decrease in prescribing is multifactorial. Per the Drug Enforcement Agency, prescription opioid sales have been declining since 2014 [50] which predates our available PDMP data. Given that PA’s increased PDMP rigor coincides with important legislative actions in 2016, we cannot directly attribute decreases in prescribing to the enhanced PDMP model and its supporting legislation. However, similar legislative actions supporting increased PDMP rigor in Ohio, Kentucky, Florida, New York, Tennessee, and Oregon over the past decade have demonstrated decreases in MME per capita and prescription opioid-related overdose deaths to varying degrees [51]. Further research is needed to understand the state policies and practices that create an optimal PDMP structure for opioid harm reduction, and we aim to contribute by transparently reporting the PDMP legislative changes and data in PA. While today’s opioid overdose deaths are driven by illicitly manufactured fentanyl nationally, reducing the prescribing rate and amount of prescription opioids remains an important strategy, particularly for opioid-naïve individuals. Additionally, omitting data on tramadol from the PA PDMP is a limitation that prevents direct comparison of opioid prescribing trends with national databases or other state PDMPs that include this controlled substance. Given that tramadol is trended on a national level and linked to a higher risk of death in certain populations [28], we recommend that PA include this metric in the available PA PDMP data. We also recommend that the PA PDMP data on opioid quantity be presented more granularly than < 21 pills. A breakdown of prescription quantities in categories such as 0–5, 6–10, 11–15, and 16–20 pills may provide important distinctions in opioid consumption from this patient group and improve prescribing practices.

## Conclusion

Based on a longitudinal review of dispensed opioid prescriptions reported to the PA PDMP database from 2016 to 2020, opioid prescribing has decreased significantly in PA. Although we cannot attribute decreased prescribing directly to the PDMP but rather to a collection of legislative actions and increased provider education, we show that PDMP data is an important tool to track the prescribing of controlled substances and measure the impact of prescription opioid harm reduction initiatives. Additionally, the implementation of mandatory PDMP reporting for controlled substances creates the potential for a nationally linked database that may enhance opioid prescription tracking. Further research is needed to compare and improve the effectiveness of different legislative PDMP policies and practices nationwide.

## Data Availability

The datasets generated and/or analyzed during the current study are available from the corresponding author on reasonable request.
